# Current Perspectives on the Importance of Pathological Features in Prognostication and Guidance of Adjuvant Chemotherapy in Colon Cancer

**DOI:** 10.3390/curroncol29030116

**Published:** 2022-02-23

**Authors:** Kabytto Chen, Henry Wang, Geoffrey Collins, Emma Hollands, Irene Yuen Jing Law, James Wei Tatt Toh

**Affiliations:** 1 Discipline of Surgery, Faculty of Medicine and Health, The University of Sydney, Sydney 2145, Australia; henry.wang@health.nsw.gov.au (H.W.); geoffrey.collins@health.nsw.gov.au (G.C.); emma.hollands@health.nsw.gov.au (E.H.); 2 Division of Colorectal Surgery, Department of Surgery, Westmead Hospital, Westmead 2145, Australia; yuenjing.law@health.nsw.gov.au

**Keywords:** PiCC UP study, colon cancer, prognosis, survival, pathological features, adjuvant therapy, chemotherapy

## Abstract

There is not a clear consensus on which pathological features and biomarkers are important in guiding prognosis and adjuvant therapy in colon cancer. The Pathology in Colon Cancer, Prognosis and Uptake of Adjuvant Therapy (PiCC UP) Australia and New Zealand questionnaire was distributed to colorectal surgeons, medical oncologists and pathologists after institutional board approval. The aim of this study was to understand current specialist attitudes towards pathological features in the prognostication of colon cancer and adjuvant therapy in stage II disease. A 5-scale Likert score was used to assess attitudes towards 23 pathological features for prognosis and 18 features for adjuvant therapy. Data were analysed using a rating scale and graded response model in item response theory (IRT) on STATA (Stata MP, version 15; StataCorp LP). One hundred and sixty-four specialists (45 oncologists, 86 surgeons and 33 pathologists) participated. Based on IRT modelling, the most important pathological features for prognosis in colon cancer were distant metastases, lymph node metastases and liver metastases. Other features seen as important were tumour rupture, involved margin, radial margin, CRM, lymphovascular invasion and grade of differentiation. Size of tumour, location, lymph node ratio and EGFR status were considered less important. The most important features in decision making for adjuvant therapy in stage II colon cancer were tumour rupture, lymphovascular invasion and microsatellite instability. BRAF status, size of tumour, location, tumour budding and tumour infiltrating lymphocytes were factored as lesser importance. Biomarkers such as CDX2, EGFR, KRAS and BRAF status present areas for further research to improve precision oncology. This study provides the most current status on the importance of pathological features in prognostication and recommendations for adjuvant therapy in Australia and New Zealand. Results of this nationwide study may be useful to help in guiding prognosis and adjuvant treatment in colon cancer.

## 1. Introduction

Colon cancer is a leading cause of cancer mortality worldwide [1]. It is a malignancy that displays a diverse set of clinicopathological features and outcomes [2,3]. Outcomes in colon cancer vary greatly; 5-year survival rates range from 10–90% depending on stage and other factors [1].

Current prognostication of colon cancer relies mainly on cancer staging defined by the Union for International Cancer Control (UICC) and American Joint Committee on Cancer (AJCC) TNM staging classification [4]. However, there are considerable differences in prognosis within patients of the same pathological stage, especially within the intermediate stages of colon cancer (stages II and III) [5,6]. Further risk stratification may be beneficial to identify patients at high risk of recurrence or metastases and to guide prognostication and subsequent cancer management. This is particularly relevant in stage II colon cancer, where prognosis is relatively good. Five year overall survival rates range from 70% to 85%, and surgery is curative in 75–80% of cases [5]. Adjuvant chemotherapy shows only an absolute survival benefit of 3–4% in stage II colon cancer patients, but up to 30% of this cohort receives adjuvant chemotherapy, which may be also associated with serious side effects that affect quality of life [6,7]. Therefore, a significant number of stage II colon cancer patients may be subjected to the side effects of chemotherapy without much benefit. More research on identifying histological and molecular markers with prognosticative ability is required to identify the high-risk cohort who would benefit from chemotherapy in stage II colon cancer.

Histological features such as tumour budding, perineural invasion, apical lymph node positivity, lymph node yield, lymph node ratio and molecular features such as microsatellite instability (MSI), KRAS, BRAF and CDX2 have been identified in their ability to guide prognostication and optimise adjuvant treatment. However, there is no clear consensus on which pathological features and biomarkers are important in guiding prognosis in colon cancer and adjuvant therapy in stage II colon cancer.

Results from the PiCC Up study were first published by Toh et al. in 2020, which focused on the role of MSI as a biomarker in colon cancer [8]. This present study uses the same dataset from the PiCC Up study previously published [8]. However, the aim of this study was to assess specialist attitudes and evaluate the importance of all the pathological features of colon cancer studied in the PiCC Up study rather than just MSI in guiding prognosis and in decision-making for adjuvant therapy for stage II colon cancer. This study interrogates the views and perspectives of key decision-makers of colon cancer multidisciplinary teams (MDTs) including colorectal surgeons, medical oncologists and gastrointestinal pathologists in Australia and New Zealand.

## 2. Materials and Methods

Data was abstracted from the original Pathology in Colon Cancer, Prognosis and Uptake of Adjuvant Therapy (PiCC UP) Australia and New Zealand study under the terms of the Creative Commons Attribution Licence (CC BY) [8]. Institutional board approval was obtained (5270) AU RED LNR/17/WMEAD/343.

The PiCC UP study was distributed to members of the Colorectal Surgical Society Australia and New Zealand (CSSANZ), Australasian Gastrointestinal Pathology Society (AGPS), and Medical Oncology Group of Australia (MOGA). One hundred and sixty-four specialists (45 oncologists, 86 surgeons and 33 pathologists) responded and participated in the survey. The survey was open for collection of data from October 2018 to January 2019. Participants who completed the questionnaire had specialist qualifications in pathology, medical oncology or colorectal surgery.

Participant attitudes for the following clinicopathological features affecting colon cancer prognostication were assessed using a 5-scale Likert score; distant metastasis; nodal involvement; tumour rupture (pT4); circumferential resection margin (CRM); lymphovascular invasion (LVI); histological grade; perineural invasion (PNI); microsatellite instability (MSI); lymph node yield (LNY); lymph node ratio (LNR); BRAF mutation; tumour location; tumour size; apical lymph node (ALN); tumour budding; KRAS mutation and CDX2 status. The 5-scale Likert score ordinal scale comprised of “not at all” (1), “not really” (2), “neutral” (3), “likely” (4) and “definitely” (5). There was also an “unsure” option.

Attitudes for the above-mentioned features affecting decision making for adjuvant chemotherapy in stage II disease were also assessed using a 5-scale Likert score. The following clinicopathological features were not included as it was not applicable in stage II disease; lymph node ratio (LNR); apical lymph nodes (ALN); distant metastasis and nodal involvement.

Descriptive statistics and weighted averages were calculated for all pathological features. Data was then analysed using a rating scale and graded response model in item response therapy model (IRT) on STATA (Stata MP, version 15; StataCorp LP, College Station, TX, USA).

As weighted means in an ordinal scale do not provide the best representation of results in this setting, IRT modelling was used to statistically analyse the results of this survey. IRT modelling is commonly used to analyse Likert-type surveys, particularly in psychometric assessments. IRT modelling scales each item based on the responses to each item and the ability of the respondents on the same metric. Thus, IRT scales the responses to the item according to the ability of respondents answering the questionnaire. The relationship between a respondent’s answer on an item and the respondent’s overall response is incorporated into scaling each individual item. Test Characteristics Curves (TCC) were generated and IRT scores were abstracted after modelling and fit of IRT analysis. In some cases, where there were too many discontinuous regions within the TCC, an IRT score would not be able to be calculated.

The obtained IRT score was then used to stratify clinicopathological features into a system used by the American College of Pathologists [9]. An IRT score of ≥4.50 was classified as Category I; between 4.00 and 4.49 as Category IIa; 3.50 and 3.99 as Category IIb; 2.50 and 3.49 as Category III; <2.50 as Category IV for prognostication. After IRT modelling, the pathological features were ranked in importance according to IRT score.

For determining adjuvant chemotherapy in stage II colon cancer, IRT scores were grouped to identify overall importance specialists placed on the pathological features. A score of ≥4.50 (rounded to 5) was considered to definitely influence decision-making for adjuvant chemotherapy for stage II colon cancer; 3.50–4.49 (rounded to 4) likely; 2.50–3.49 (rounded to 3) neutral; <2.5 (rounded to 1–2) not likely to be useful in guiding adjuvant therapy.

A subgroup analysis analysing attitudes within each specialty (surgeons, pathologists and medical oncologists) was performed to detect different priorities each specialty placed on each pathological feature. A mean score and IRT score were calculated, and a Kruskal–Wallis test was used to test the difference between the three groups.

## 3. Results

One hundred and sixty-four specialists (45 oncologists, 86 surgeons and 33 pathologists) participated in the survey. Of the participants, 80.5% regularly participated in colorectal MDTs. Six advanced trainees (non-specialists) responded to the questionnaire, and were excluded from analysis.

A descriptive overview of specialist opinions on pathological features influencing prognostications is detailed in Table 1. Highest ranked features included distant metastasis, tumour rupture, lymph node involvement and involved surgical margin. CDX2 status, EGFR status, tumour size, tumour location and lymph node ratio (LNR) were ranked of lowest importance in determining prognosis in colon cancer.

A descriptive overview of specialist opinions on pathological features influencing adjuvant treatment in stage II colon cancer is detailed Table 2. Highest ranked features included tumour rupture, surgical margin involvement, LVI, positive circumferential margin and perineural invasion. CDX2 status, tumour location, tumour size, TILs, KRAS and BRAF status were ranked of lowest importance in determining prognosis in colon cancer.

Pathological features that specialists were most unsure of included CDX2 status, EGFR status, Lymph node ratio, KRAS status, BRAF status and tumour budding. IRT modelling was achieved in 17 pathological features for prognosis, detailed in Table 3. IRT scores ranged from 2.91 to 4.88. Nine pathological features were classified into grade I level of evidence (IRT score ≥ 4.50), four into grade IIa (IRT score 4.00–4.49), two into grade IIb (IRT score 3.50–3.99) and two into group III (IRT score < 2.50).

IRT modelling was achieved in 10 pathological features for adjuvant therapy, detailed in Table 4. IRT scores ranged from 2.24 to 4.55. There was a much wider variation in clinician perspectives towards pathological features in determining adjuvant therapy in stage II colon cancer compared to prognostication. Features that were highly ranked included tumour rupture, LVI and MSI-H. A large proportion of clinicians did not believe tumour location or tumour size to be of use in determining adjuvant therapy for stage II colon cancer.

## 4. Discussion

Understanding molecular and histological pathological features in colon cancer with prognosticative ability may help identify colon cancer patients at higher risk of recurrence or metastasis, allowing more accurate prognostication and guidance with adjuvant therapy. Currently, TNM staging by Union for International Cancer Control (UICC) and American Joint Committee on Cancer (AJCC) TNM is the main driver of prognostication [2,5]. However, there are also histological and molecular markers that may be useful in prognostication and optimisation of adjuvant treatment. Histological features include tumour budding, perineural invasion, apical lymph node positivity, lymph node yield and lymph node ratio. Molecular features include MSI, KRAS, BRAF and CDX2 [3,4,10,11,12,13,14]. Currently, there is no international consensus on their roles, and different international guidelines utilise different pathological features to help with prognostication and decision-making in adjuvant chemotherapy in stage II colon cancer [5,6].

To date, this survey was the largest survey of specialists in Asia Pacific on the attitudes regarding different pathological features in guiding prognosis and adjuvant treatment in colon cancer. By comparing current perspectives with current evidence in literature, this study identified that a significant number of specialists were unsure of the role of several biomarkers such as CDX2, EGFR, KRAS and BRAF status on the prognostication of colorectal cancer and its influence on adjuvant therapy in stage II disease. Specialists had more awareness of MSI. We have previously published on the importance of MSI using the PiCC Up data [8] as well as a comprehensive review of the literature on the value of pathological features in guiding prognostication in colorectal cancer [15]. A better understanding of molecular markers and pathological features is important in the move towards precision oncology, with molecular markers and pathological features used to guide prognostication and identification of high-risk stage II colon cancer patients for chemotherapy as well as other management decision-making strategies.

In this discussion, we compare the results of this survey with the evidence in the existing literature, identifying the gaps in knowledge, barriers and limitations of the current evidence as well providing an understanding of where the strength of evidence lies. Features have been grouped together based on the grading system based on IRT scores (see Table 3).

### 4.1. Grade 1 Features

#### 4.1.1. Distant Metastasis, Lymph Node Metastasis and Tumour Rupture

In keeping with existing literature, TNM staging was seen as the most important consideration in prognostication of colon cancer by Australian and New Zealand specialists. Presence of distant metastasis, lymph node metastasis and advanced tumour stage obtained highest scores on IRT modelling and all clinicians were aware of its prognosticative significance. The AJCC UICC TNM staging criteria is used widely in international guidelines [2,3,4,11,14]. Distant metastasis and regional nodal involvement are the strongest predictors of poor prognosis [4,16]. Distant metastasis confers a 10–15% 5 year survival rate [17]. Nodal involvement also decreases five year survival significantly (30–60% vs. 70–90%, node positive vs. node negative) [18,19], and chances of cancer recurrence are higher (30–35%) [20,21]. Similarly, higher T-staging also has a significant negative prognostic effect, being associated with both nodal and metastatic spread. Within T4 tumours, some may also be unresectable, and attempts at resection may lead to R1 or R2 incomplete resections [22].

This study also found tumour rupture (advanced T-stage) to “definitely” influence decision making, in line with international guidelines. The American Society of Clinical Oncology (ASCO), National Comprehensive Cancer network (NCCN), American Society of Colon and Rectal Surgeons (ASCRS), European Society for Medical Oncology (ESMO) and National Health and Medical Research Council (NHMRC) guidelines all identify tumour perforation as a high-risk feature that when considering adjuvant chemotherapy in stage II colon cancer [3,5,10,12,14].

#### 4.1.2. Circumferential Resection Margin (CRM)

In this study, Australian and New Zealand specialists identified CRM as an important histological consideration in guiding prognosis and need for adjuvant therapy ranking 5 (of 18) in order of importance in the decision-making for adjuvant therapy. This is similar to international guidelines such as NCCN and ESMO, where CRM positivity represents a pathological feature within its criteria for adjuvant chemotherapy in stage II colon cancer [3,10]. It is included in standardised histological reporting criteria for colorectal cancers [23,24]. CRM greater than 1 mm is considered negative and is recommended for all resections. CRM positivity may correlate with the quality of surgery performed [3,10,12,13]. Occurring in up to 20% of cases, CRM positivity is associated with advanced stage on initial diagnosis and high-risk histological features such as perineural (PNI) and lymphovascular invasion (LVI). It is a strong indicator of recurrence and reduced survival in colorectal cancers [25,26].

#### 4.1.3. Lymphovascular Invasion (LVI)

LVI is considered a key component in the progression to lymph node metastasis and is well recognised as a poor prognosticator in colon cancer, independent of stage [27,28,29,30,31,32,33]. Patients with LVI positivity have an increased risk of overall mortality and a lower disease-free survival (DFS) [27,30,31,32,33]. The AJCC-UICC TNM staging manual currently classifies LVI as a factor in the prognostication of CRC [4], and recommends routine pathological assessment [24]. While Australian guidelines do not specifically identify LVI in determining need for adjuvant therapy, our survey shows an awareness of LVI as a factor in influencing adjuvant therapy in stage II colon cancer among Australian and New Zealand specialists. LVI ranked 8 (of 23) in order of importance for prognostication and 8 (of 17) after IRT modelling.

LVI also ranked second (of 10) in IRT modelling in importance when influencing adjuvant therapy decision making. Specialists in our survey thought LVI was “likely” to affect adjuvant therapy. This is reflective of multiple international guidelines, with LVI included in the definition of “high-risk” stage II colon cancer in the ASCO, NCCN and ESMO guidelines [3,10,14]. Adjuvant chemotherapy may have a protective effect in the setting of Stage II colon cancers and LVI positivity, with Gao et al. demonstrating improved 5-year overall survival (OS) (66.7% vs. 40.9%, *p =* 0.004) and DFS rates (64.1% vs. 36.3% *p =* 0.002) [28]. However, there is also evidence in the literature suggesting that adjuvant chemotherapy may not improve survival in stage II colon cancer patients with high risk factors [6,34,35]. Further randomised controlled trials selectively targeting high-risk patients will need to be conducted in the future.

#### 4.1.4. Histological Grade

Our study showed that histological grade is likely to be an important marker of prognosis with the majority of specialists indicating that it was likely to influence prognosis in colon cancer. Tumour grade has been consistently reported to be a stage-independent negative prognostic factor in colon cancer [36,37,38,39]. It is utilised in most international guidelines and documentation is standard in specimen assessment. The latest edition of the AJCC TNM staging system identifies histological grade as a prognostic factor in colon cancer [4,23]. A lack of a standardised system and interobserver variability remains the main limitation in the assessment of tumour grading [36,38,40]. Patients with high grade colon cancer have an increased recurrence risk with a lower DFS and disease specific survival (DSS) compared to those with low grade disease [9,38,39,41,42]. High grade early stage I and II colon cancer may have worse prognosis than low grade stage III disease in terms of DSS [40].

The majority of survey respondents (62%) also believed that it would influence recommendations for adjuvant therapy. In stage II disease, a high tumour grade is identified as a high-risk factor for recurrence by many international guidelines, with NCCN, ASCO, ESMO, ASCRS, NHMRC and NCI guidelines recommending adjuvant chemotherapy in this subset of patients [3,10,11,12,13,14].

### 4.2. Grade IIa Features

#### 4.2.1. Perineural Invasion

A majority of specialists believed that PNI status was likely to influence prognosis in colon cancer (89%). PNI was ranked similar to MSI status and LVI in this study. In the literature, PNI is considered on par with other established prognostic factors such as tumour grade, T-stage, N-stage, metastasis and extramural invasion [43]. Colon cancer patients with PNI have significantly worse OS, DFS and CSS, independent of cancer stage [43,44,45,46,47]. PNI is identified as an additional tumour related prognostic factor by the latest AJCC-UICC TNM staging criteria [4].

A majority of specialists also believed that PNI status was likely to guide recommendation for the use of adjuvant therapy in stage II disease (70%). In the literature, several authors have suggested the use of PNI status to facilitate the selection of patients with stage II colon cancer for adjuvant chemotherapy [5,6,48,49]. Liebig et al. reported that stage II PNI-positive patients had poorer prognosis compared to stage III PNI-negative patients [45]. Multiple international oncology guidelines (National Comprehensive Cancer Network (NCCN), ASCRS American society of colon and rectal surgeons (ASCRS) and European Society for Medical Oncology (ESMO)) utilise perineural invasion as a risk factor for recurrence and recommend consideration of adjuvant chemotherapy in this subset of patients [3,5,10].

#### 4.2.2. Microsatellite Instability (MSI)

The role of MSI in colon cancer by the PiCC Up study has been explored in an earlier study by Toh et al. (2020) [8]. MSI was highlighted as an important biomarker in prognostication in this study. It was ranked more important than KRAS, BRAF and CDX2 and was ranked similar to LVI and PNI. MSI-H has been shown to convey a better prognosis in early stage CRC [50,51,52], and resistance to cytotoxic chemotherapy in mCRC [12].

MSI was also considered important in guiding adjuvant therapy in stage II colon cancer in this survey. Specialists believed MSI was “likely” to influence decision making for adjuvant therapy in stage II colon cancer. MSI status may be useful in guiding adjuvant chemotherapy in stage II colon cancer [3]. MSI-H has been shown to have a protective effect in stage II/III colon cancer in a study by Sargent et al. and a more recent meta-analysis by Bockelman et al. [53,54]. As a result, stage II colon cancer patients with MSI-H tumours may be less likely to benefit from chemotherapy [54]. Furthermore, MSI has been shown to be important in the management of metastatic colon cancer as it selects patients who would benefit from immunotherapy as a second line agent [11,12,55].

#### 4.2.3. Lymph Node Yield (LNY)

A majority of colorectal cancer specialists identified lymph node yield (LNY) as a pathological feature of prognostic importance in this survey (81%). LNY ranked 13 (of 17) in order of importance after IRT modelling. In the existing literature, LNY may be a strong prognostic factor, particularly for stage I-III colon cancer [56,57,58,59,60,61]. Improved prognosis independent of nodal stage has been observed in colon cancer with a higher LNY in terms of recurrence, DFS and mortality [57,58,60,61,62,63]. International guidelines recommend a LNY ≥ 12 for adequate nodal staging [3,4,11,12,24,64].

In stage II colon cancer, 54% of clinicians believed LNY influences chemotherapy decision making, with an overall “neutral” view on IRT modelling. There is strong evidence that a low LNY increases the risk of recurrence [3,65]. LNY < 12 is identified as a major prognostic factor in stage II disease for determining adjuvant chemotherapy in ESMO and NCCN guidelines [3,10].

#### 4.2.4. BRAF Status

In this study, 73% of specialists identified BRAF as “likely” to or “definitely” influence prognosis. Within the literature, the prognosticative utility of BRAF remains controversial [66,67,68,69,70]. Of all BRAF mutations, the BRAF V600E mutation accounts for the majority (approximately 90%) in colon cancer [71]. Current evidence leans towards poor prognostication in BRAF V600E-mt disease, though its prognosticative value may vary according to stage and MSI status [69,70,72,73,74,75,76]. In stage II and III colon cancer, studies have shown worse prognosis in BRAF V600E-mt patients, with increased mortality and reduced PFS [66,77]. In metastatic disease, BRAF V600E-mt confers resistance to EGFR inhibitor therapy [78,79,80,81,82]. The prognosticative ability of BRAF V600E may be augmented by the combination of other biomarker tests (PIK3CA, KRAS and NRAS) [83]. In the non-metastatic setting, testing for BRAF is recommended in patients with MSI-H colon cancer to identify those with Lynch syndrome; there is no current role in determining prognostication [3,11,84]. In metastatic colon cancer however, NICE (2020), ESMO (2014) and Australian (2017) guidelines highlight a role for BRAF testing for prognostication and prediction of resistance to anti-EGFR therapies [12,55,64].

Within our survey, BRAF was not seen as an important factor in guiding adjuvant therapy. BRAF ranked the 5th most common pathological feature in which clinicians did not know if it affected prognosis or adjuvant therapy. The evidence for BRAF V600E as a biomarker in guiding recommendations for adjuvant chemotherapy in stage II colon cancer is scant. A 2011 study by Hutchins et al. looked 1913 patients from the QUASAR trial found no significant difference in recurrence risk between BRAF-mt and BRAF-wt patients, and did not identify BRAF as a useful biomarker to guide the use of chemotherapy [62].

### 4.3. Grade IIb Features

#### 4.3.1. Lymph Node Ratio (LNR)

In this study, approximately two thirds of specialists believed that LNR “likely” or “definitely” influences prognosis. High LNR independently predicts reduced OS and DFS in stage III and metastatic disease [85,86,87,88,89,90,91,92]. Several systematic reviews and meta-analyses have demonstrated improved OS (2.36) and DFS (3.71) in colon cancer patients with lower LNR [85,86]. In addition, LNR has superior predictive capability compared to current N-staging (number of positive nodes) in some studies [85,86,90]. The prognostic significance of LNR may be greater where LNY is <12 [88,93].

Current AJCC-UICC TNM staging does not consider LNR part of N-staging [4]. While several studies have demonstrated the prognosticative ability of LNR, it is not utilised in any international colon cancer guidelines [3,10,11,12,13,55,64]. Further research is required.

#### 4.3.2. Tumour Location

In this study, tumour location ranked fourth last (of 23) in importance. However, recent literature has shown some potential utility in the prognostication of colon cancer. Right sided CRC have a greater tendency to metastasise to the peritoneal surface compared to left sided CRC which metastasises more frequently to liver and lung [94,95]. In the non-metastatic setting, there may be some utility in prognostication, but the level of evidence is weak [96,97,98,99,100,101]. International guidelines do not consider tumour location as a major factor driving prognostication or management in non-metastatic CRC [3,11,13,14].

In this study, tumour location ranks last in IRT analysis, with the consensus that tumour location should not be used to guide adjuvant therapy in stage II colon cancer. There is sparse evidence in the literature that tumour location may influence adjuvant therapy. Tumour sidedness is not factored in any international guideline in determining adjuvant therapy for stage II colon cancer [5,6].

### 4.4. Grade III Features

#### Tumour Size

In this study, most specialists did not believe tumour size would influence prognosis. Tumour size was ranked second last in order of importance after IRT analysis. This is in line with the existing literature, which highlights an inconclusive role of tumour size in prognostication in colon cancer, despite its established utility in malignancies such as thyroid, breast and lung cancer [4]. Some studies have reported a worse prognosis with increasing tumour size in colon cancer [102,103,104,105,106,107,108,109]. Authors have postulated that inherent difficulties in achieving a complete R0 resection secondary due to its size or the vertical invasion mechanics of the tumour could potentially explain the survival difference [102]. Other studies have failed to find independent prognosticative ability of tumour size in colon cancer [9,110,111,112,113,114,115,116]. Significant heterogeneity within the literature is due to different tumour size cut-off values used in different studies, and defining a consensus cut-off is a direction for future research [117]. While histological reporting routinely record tumour size, it is not utilised in current TNM staging [4] or considered in routine management of colon malignancy [3,10,14,55,64].

This study demonstrated that majority of specialists do not believe that tumour size influences decision making for adjuvant therapy in stage II colon cancer in our study. In the literature, tumour size is not included in any guidelines regarding colon cancer.

### 4.5. Other Features

#### 4.5.1. Apical Lymph Nodes (ALN)

In our study, a large proportion of specialists were aware of ALN and a majority of those believed it did influence prognostication in colon cancer. The impact of ALN involvement on prognostication is controversial [118,119,120,121,122]. Some authors have shown a positive ALN to independently predict poorer OS and DFS in colon cancer patients, with potentially better predictive capability than current N staging [120,121,123,124]. Other studies found that survival outcomes in patients with stage II disease and ALN involvement were similar to those with stage IV disease with R0 resection. As a result, some authors have argued that ALN metastasis reflected systematic involvement as opposed to regional spread [121,125]. On the other hand, several studies have not found such an association, suggesting that ALN involvement should be still seen as a regional metastasis rather than systemic metastases [118,119,122,126]. Currently there is no set consensus for the definition of ALN metastasis [127]. Current TNM nodal staging considers ALN as a regional lymph rather than a separate entity [4]. However, ALN nodal involvement is currently utilised in the current Australian and Japanese classification system for colon cancer [12,13]. ALN positivity (previously classified as N3) has been previously utilised in previous editions of the TNM staging system (3rd and 4th edition), but complexity in dividing lymph node zones for pathologic evaluation and inconsistent evidence in prognostication have led to its disuse in future editions [123].

ALN is not applicable in decision making for adjuvant therapy in stage II colon cancer as ALN positivity denotes at least stage III disease [4].

#### 4.5.2. Tumour Budding

In this study, a significant proportion of specialists were unsure of the role of tumour budding in prognostication of colorectal cancer. This may be due to the fact that tumour budding is a less well-known pathological feature. In the literature, tumour budding has emerged as an independent prognostic biomarker in colon cancer [128]. Strong evidence has linked the presence of tumour budding with reduced OS and DFS in colon cancer [128,129,130,131]. Its utility is highest in early disease (stage I and II) due to its potential to identify patients at high risk of nodal dissemination [128,132,133]. In the last few years, tumour budding has been acknowledged as a potential tumour-related prognostic factor in latest AJCC-UICC TNM classification system [4]. Latest UK, European and Japanese guidelines include tumour budding in the screening, diagnosis and treatment of colon cancer [3,4,55,64,134] as a high-risk feature.

Forty-eight percent of specialists in this survey believed that tumour budding may influence decision making for adjuvant therapy in stage II colon cancer. On IRT modelling, tumour budding had an overall “neutral” ranking in influencing adjuvant therapy in stage II colon cancer. In the literature, the role for tumour budding to identify high risk stage II colon cancer patients who may benefit from adjuvant chemotherapy has been reported but not extensively studied [129,130,135,136]. Overall, tumour budding may represent a promising histological feature in influencing decision making for stage II colon cancer.

#### 4.5.3. Tumour Infiltrating Lymphocytes (TILs)

The majority (80%) of survey participants believed TILs to influence prognosis in colon cancer. This is consistent with the existing literature, where a high TIL count has shown to be associated with a better prognosis. Higher OS, CSS and DFS have been observed in colon cancer patients with higher density of TILs [137,138]. Subtypes of TILs shown to have greatest prognosticative ability include CD3, CD8 and FoxP3 cells [138,139]. Currently, assessment of TILs is not commonly used for prognostication in the clinical environment [3,10,11,12,14,64]. Recent updates to international guidelines have acknowledged the potential role of immunoclassification scores such as the Immunoscore (which include TIL assessment) alongside established TNM staging to guide prognostication and recurrence prediction in colon cancer [3,140].

Regarding guiding adjuvant therapy in stage II colon cancer, only a minority (30%) of specialists believed that TILs would influence decision making. A small proportion of respondents (5.93%) were unsure of the effect of TILs on adjuvant therapy. On IRT modelling, overall attitude towards TILs was “neutral”, ranking TILs similar to BRAF status and tumour budding in guiding adjuvant chemotherapy. Current Australian colon cancer guidelines do not incorporate TILs into prognostication or adjuvant chemotherapy in stage II colon cancer management [12]. Some authors have highlighted the potential of TILs to guide decision making in adjuvant chemotherapy and immunotherapy [138,139]. ESMO guidelines discuss the role of scoring systems incorporating TIL counts (such as the Immunoscore) in determining recurrence risk in challenging cases of stage II colon cancer [3].

#### 4.5.4. KRAS Status

In our study, KRAS did not rank high (18 of 23) in importance for guiding prognosis in colon cancer. This is in keeping with current literature, which has not definitively established KRAS mutation as an independent prognostic factor, especially in stage II and III colon cancer [141]. KRAS testing appears to be most useful in metastatic colon cancer and may be helpful in guiding responsiveness to anti-EGFR targeted therapy. In metastatic colon cancer, KRAS-mt has been associated with poorer prognosis, with lower OS and RFS compared to the KRAS-wt population [142,143,144,145]. A pooled analysis by Modest et al. of 1239 mCRC patients demonstrated that KRAS-mt patients had a reduced OS (HR 1.41, *p* < 0.001) and PFS (HR 1.2, *p* = 0.03) compared to the KRAS-wt cohort [82]. Evidence is less definitive in non-metastatic colon cancer. Multiple phase III chemotherapy trials have found an association between KRAS and poorer OS, DFS as well as an increased risk of recurrence [146,147,148,149]. However, other studies have reported KRAS to have no prognostic influence [146,150,151,152]. Currently KRAS mutation is considered an additional prognostic factor in AJCC-UICC staging [4].

In this study, only 31% of specialists believed KRAS would influence decision making for adjuvant chemotherapy in stage II disease. In the existing literature and established guidelines, routine testing of KRAS is not recommended in the workup of non-metastatic colon cancer due to a lack of evidence in its utility for determination of adjuvant therapy [3,12,64].

#### 4.5.5. CDX2 Status

Our study found a significant uncertainty amongst specialists regarding CDX2 and its influence on prognosis and adjuvant therapy. A large percentage of specialists (42%) were unsure if CDX2 affected prognosis. Traditionally used in identifying cancers of unknown origin [153], CDX2 has recently been identified as an emerging prognostic biomarker in colon cancer. Recent level 1 evidence highlights the association of a loss of CDX2 expression with worse prognostication in colon cancer (reduced OS and DFS) [154,155,156]. A recent systematic review and meta-analysis revealed that CDX2 expression reduced mortality, with a 50% reduction in death compared to patients with colorectal cancer with poor or no CDX2 expression. This association was more pronounced in stage II and III colon cancer, with up to 70% risk reduction in OS. CDX2 expression was also associated with up to a 52% lower risk of disease recurrence [155]. While some studies have demonstrated the association between CDX2 loss and negative prognosis, there are inconsistencies within the literature. Bruun et al. demonstrated that CDX2 was prognostic only in stage IV and stage III BRAF-mutated colon cancer patients [157]. Silk et al. showed that CDX2 loss was associated with worse prognosis only in the MSS cohort but not the MSI-H cohort in stage II colon cancer [158]. Other authors have failed to find an association between CDX2 and prognosis [15,159,160]. Currently CDX2 is not utilised in prognostication in colon cancer in the clinical setting. Current guidelines including NICE, NCCN, Australian and ESMO do not report CDX2 as a useful tool for prognostication [3,10,12,55,64].

A large proportion of specialists (32%) were also unsure of the effect of CDX2 on adjuvant therapy. CDX2 ranked last in order of importance in affecting adjuvant therapy; the majority of specialists did not believe it had any value in prognosis or decision making for adjuvant chemotherapy. Currently, there is minimal literature on CDX2 influencing adjuvant therapy in stage II colon cancer and its role in prognostication has not yet been established [159].

#### 4.5.6. EGFR Status

There was also uncertainty amongst specialists regarding EGFR and its influence on prognosis and adjuvant therapy. EGFR is commonly over expressed in colorectal cancer on immunohistochemical analysis; however, it has not been established as an independent prognostic variable [161]. Although rare in primary CRC [162], EGFR extracellular domain (ECD) mutations have been demonstrated in patients with mCRC treated with anti-EGFR therapy [163,164]. Patients with more prolonged responses to EGFR therapy preferentially develop EGFR ECD mutations compared to RAS mutations. These mutations are associated with the clonal evolution of drug-resistant cancer cells in patients treated with anti-EGFR antibodies. EGFR mutation testing is not routinely performed in primary CRC and its utility in mCRC is uncertain.

### 4.6. Limitations of Study

This study has some limitations. Firstly, because the PiCC UP study is a survey designed to assess the attitudes of specialist clinicians, this study does not provide the same level of evidence from clinical data seen in randomised control trials or cohort studies. Nonetheless, this survey to date was the largest survey of specialists in the Asia Pacific on the importance on different pathological features of colon cancer in guiding prognosis and adjuvant treatment. Another limitation was that due to the complex modelling required in IRT analysis, IRT scores was not computable for six pathological features in prognostication and eight pathological features for adjuvant therapy in stage II disease. However, IRT analysis is a commonly used form of analysis for survey-based studies that incorporates sophisticated mathematically modelling to analyse survey data. The IRT results in this study provided good correlation with both the weighted mean as well as Likert plot analysis reported in this study.

## 5. Conclusions

This study provides the most current perspectives by specialists on the importance of pathological features in prognostication and recommendations for adjuvant therapy in Australia and New Zealand. There is greater variation in specialist perspectives in recommendation for adjuvant therapy in stage II colon cancer than prognostication. In this study, the most important pathological features in prognostication were distant metastasis, lymph node metastases, tumour rupture, involved surgical margin, positive circumferential resection margin, lymphovascular invasion and histological grade. The pathological features that were considered important in guiding adjuvant therapy were tumour rupture, lymphovascular invasion and microsatellite instability. IHC markers such as CDX2, EGFR, KRAS and BRAF status present areas for further research to improve precision oncology. This is the largest survey of Australia and New Zealand specialists on pathological features in colon cancer and understanding the perspectives of key stakeholders in colon cancer management may be useful in informing future guidelines.

## Figures and Tables

**Table 1:** Likert score table for specialist responses to whether pathological feature influence prognostication. Adapted from Wiley Cancer Reports—Toh et al. (2020) [8] under the creative commons attribution license (CC BY).

Pathological Feature	Not at All	Not Really	Neutral	Likely	Definitely	Don’t Know
Distant metastases—extra hepatic	0.00%	0.00%	0.00%	**4.41%**	**94.12%**	1.47%
Tumour rupture (pT4)	0.00%	0.00%	1.47%	**10.29%**	**88.24%**	0.00%
Involved lymph nodes	0.00%	0.74%	0.74%	**6.62%**	**91.18%**	0.74%
Distant metastases—liver	0.74%	0.00%	0.74%	**7.35%**	**89.71%**	1.47%
Involved surgical margin	0.00%	0.00%	1.48%	**10.37%**	**86.67%**	1.48%
Circumferential resection margin	0.00%	1.47%	4.41%	**14.71%**	**77.94%**	1.47%
Involved radial margin	0.00%	1.48%	4.44%	**8.15%**	**82.96%**	2.96%
Lymphovascular invasion	0.74%	0.74%	2.22%	**25.93%**	**69.63%**	0.74%
Invasion beyond muscularis propria	0.00%	4.44%	2.22%	**28.15%**	**65.19%**	0.00%
Grade (degree of differentiation)	1.47%	3.68%	2.21%	**27.94%**	**64.71%**	0.00%
Microsatellite instability (MSI)	0.00%	1.48%	6.67%	**27.41%**	**62.22%**	2.22%
Perineural invasion (PNI)	0.75%	2.24%	6.72%	**38.81%**	**50.00%**	1.49%
Lymph node yield (LNY)	0.00%	5.19%	11.85%	**39.26%**	**42.22%**	1.48%
Apical node status	0.00%	4.44%	6.67%	**40.74%**	**44.44%**	3.70%
Tumour infiltrating Lymphocytes	0.73%	5.11%	11.68%	**48.18%**	**31.39%**	2.92%
Tumour budding	0.74%	5.88%	11.76%	**39.71%**	**36.03%**	5.88%
BRAF status	0.74%	2.22%	14.07%	**29.63%**	**43.70%**	9.63%
KRAS status	1.47%	5.15%	16.91%	**29.41%**	**36.76%**	10.29%
Right versus left side	5.15%	17.65%	16.18%	**36.03%**	**23.53%**	1.47%
Lymph node ratio (LNR)	0.74%	4.41%	14.71%	**34.56%**	**30.88%**	14.71%
Size of tumour	5.88%	**27.21%**	19.85%	**31.62%**	15.44%	0.00%
EGFR status	2.96%	8.89%	**23.70%**	**28.15%**	18.52%	17.78%
CDX2 status	4.48%	9.70%	**22.39%**	16.42%	5.22%	**41.79%**

**Table 2:** Likert score table for specialist responses to whether pathological feature influence decision-making in guiding adjuvant treatment in stage II colon cancer. Adapted from Wiley Cancer Reports—Toh et al. (2020) [8] under the creative commons attribution license (CC BY).

Pathological Feature	Not at All	Not Really	Neutral	Likely	Definitely	Don’t Know
Tumour rupture (pT4)	0.00%	0.74%	2.94%	30.15%	**64.71%**	1.47%
Involved surgical margin	2.99%	2.99%	5.97%	22.39%	**64.93%**	0.75%
Involved radial margin	3.70%	2.96%	8.15%	22.22%	**60.74%**	2.22%
Lymphovascular invasion (LVI)	1.48%	2.22%	8.89%	45.19%	**41.48%**	0.74%
Circumferential resection margin (CRM)	3.70%	8.15%	13.33%	28.15%	**45.19%**	1.48%
Perineural invasion (PNI)	1.48%	11.11%	15.56%	42.96%	**27.41%**	1.48%
Grade (degree of differentiation)	3.70%	8.89%	22.22%	38.52%	**23.70%**	2.96%
Microsatellite instability (MSI)	5.19%	15.56%	17.04%	25.19%	**34.07%**	2.96%
Lymph node yield (LNY)	5.93%	14.81%	21.48%	34.07%	**20.00%**	3.70%
Invasion beyond muscularis propria	10.53%	16.54%	19.55%	29.32%	**22.56%**	1.50%
Tumour budding	7.41%	16.30%	21.48%	35.56%	**12.59%**	6.67%
Tumour infiltrating lymphocytes (TILS)	8.89%	24.44%	30.37%	24.44%	**5.93%**	5.93%
BRAF status	10.45%	13.43%	25.37%	22.39%	**14.18%**	14.18%
KRAS status	12.69%	18.66%	25.37%	17.16%	**13.43%**	12.69%
EGFR status	13.53%	18.05%	28.57%	15.04%	**9.02%**	15.79%
Size of tumour	24.44%	34.07%	23.70%	11.11%	**3.70%**	2.96%
Right versus left colon cancer	23.13%	30.60%	29.85%	8.21%	**3.73%**	4.48%
CDX2 status	14.39%	13.64%	29.55%	8.33%	**2.27%**	31.82%

**Table 3:** Item Response Theory (IRT) and weighted average scores for pathological features influencing prognosis in colon cancer: Adapted from Wiley Cancer Reports—Toh et al. (2020) [8] under the creative commons attribution license (CC BY).

Pathological Features	Grade	Prognosis IRT Score	Lower Limit 95% C.I.	Upper Limit 95% C.I.	Weighted Average	% Likely/Definitely to Influence Prognosis
Distant Metastases	Grade I	4.88	4.85	4.91	4.88	98.53%
Lymph Node Metastases		4.88	4.56	4.96	4.86	97.80%
Tumour Rupture		4.87	4.78	4.93	4.87	98.53%
Liver Metastases		4.85	4.45	4.95	4.81	97.06%
Involved Margin		4.83	4.46	4.93	4.79	97.04%
Radial Margin		4.69	4.13	4.87	4.64	91.11%
Circumferential Resection Margin		4.65	4.63	4.67	4.65	92.65%
Lymphovascular Invasion		4.64	4.28	4.78	4.61	95.56%
Grade of Differentiation		4.52	4.35	4.63	4.51	92.65%
Microsatellite Instability	Grade IIa	4.47	4.05	4.68	4.44	89.63%
Perineural Invasion		4.35	3.77	4.59	4.31	88.81%
BRAF Status		4.3	0.211	4.95	3.84	73.33%
Lymph Node Yield		4.14	4.11	4.17	4.14	81.48%
Lymph Node Ratio	Grade IIb	3.96	0.161	4.83	3.46	65.44%
Location—Right vs. Left		3.54	2.63	4.15	3.51	59.56%
Size of Tumour	Grade III	3.23	2.93	3.52	3.24	47.06%
EGFR Status		2.97	2.58	3.31	2.97	46.67%

**Table 4:** Item Response Theory (IRT) and weighted average scores for pathological features influencing decision-making for adjuvant chemotherapy in stage II colon cancer: Adapted from Wiley Cancer Reports—Toh et al. (2020) [8] under the creative commons attribution license (CC BY).

Pathological Features	Adjuvant Chemotherapy IRT Score	Lower Limit 95% C.I.	Upper Limit 95% C.I.	Overall Recommendation	Weighted Average	% Likely/Definitively to Influence Adjuvant Treatment in Stage II Colon Cancer
Tumour Rupture	4.55	4.49	4.59	Definitely	4.54	94.86%
Lymphovascular Invasion	4.25	3.72	4.51	Likely	4.21	86.67%
Microsatellite Instability	3.62	2.89	4.15		3.59	59.26%
Lymph Node Yield	3.36	3.14	3.56	Neutral	3.36	54.07%
Invasion beyond Muscularis Propria	3.34	2.36	4.07		3.32	51.88%
Tumour Budding	3.18	1.85	3.97		3.1	48.15%
Tumour Infiltrating Lymphocytes	2.81	1.78	3.62	Neutral	2.76	30.37%
BRAF Status	2.78	1.52	3.76		2.74	36.57%
Size of Tumour	2.25	1.77	2.81	Not really	2.27	14.81%
Location—Right vs. Left	2.24	2.14	2.36		2.25	11.94%

## Data Availability

The data presented in this study are available on request from the corresponding author.

## Abbreviations

AJCC	American Joint Committee on Cancer
ALN	Apical Lymph Node
ASCO	American Society of Clinical Oncology
ASCRS	American Society of Colon and Rectal Surgeons
BRAF	B-raf
CDX2	Caudal Type Homeobox 2
CI	confidence interval
CRC	colorectal cancer
CRM	circumferential resection margin
CSS	cancer specific survival
DFS	disease free survival
EGFR	epidermal growth factor receptor
ESMO	European Society for Medical Oncology
HR	hazard ratio
IHC	immunohistochemistry
IRT	item response theory
KRAS	Kirsten rat sarcoma virus
LNR	lymph node ratio
LNY	lymph node yield
LVI	lymphovascular invasion
mCRC	metastatic colorectal cancer
MDT	multidisciplinary team
MSI	microsatellite instability
MSI-H	MSI high
NCCN	National Comprehensive Cancer Network
NCI	National Cancer Institute
NHMRC	National Health and Medical Research Council (Australia)
NICE	National Institute for Health and Care Excellence
OS	overall survival
PNI	perineural invasion
RFS	recurrence free survival
TILs	tumour infiltrating lymphocytes
TNM	Tumour, Node, Metastasis
UICC	Union for international Cancer Control
